# A Community-Based Assessment of Knowledge and Practice of Breast Self-Examination and Prevalence of Breast Disease in Southwest Cameroon

**DOI:** 10.1155/2019/2928901

**Published:** 2019-01-01

**Authors:** Kareen Azemfac, S. Ariane Christie, Melissa M. Carvalho, Theophile Nana, Ahmed N. Fonje, Gregory Halle-Ekane, Rochelle Dicker, Alain Chichom-Mefire, Catherine Juillard

**Affiliations:** ^1^Faculty of Health Sciences, University of Buea, Buea, Cameroon; ^2^Center for Global Surgical Studies, Department of Surgery, University of California, San Francisco, San Francisco, CA, USA; ^3^Department of Surgery, Regional Hospital Limbe, Limbe, Southwest Region, Cameroon; ^4^Department of Surgical Critical Care, University of California Los Angeles, Los Angeles, CA, USA

## Abstract

**Introduction:**

Despite the rising trend in breast cancer incidence and mortality across Sub-Saharan Africa, there remains a critical knowledge gap about the burden and patterns of breast disease and breast cancer screening practices at the population level. This study aimed to identify socioeconomic factors associated with knowledge and practice of breast self-examination (BSE) as well as assess the prevalence of breast disease symptoms among a mixed urban-rural population of women in the Southwest region of Cameroon.

**Methods:**

We conducted a household-level community-based study in Southwest Cameroon between January and March 2017, using a three-stage cluster sampling framework. We surveyed 1287 households and collected self-reported data on 4208 female subjects, 790 of whom were household representatives. Each household representative provided information on behalf of all female household members about any ongoing breast disease symptoms. Moreover, female household representatives were questioned about their own knowledge and practice of BSE.

**Results:**

Women demonstrated low frequency of knowledge of BSE, as 25% (n=201) of household representatives reported any knowledge of BSE; and among these only 15% (n=30) practiced BSE on a monthly basis. Age (aOR: 1.04), usage of Liquid Petroleum Gas fuel, a marker of higher socioeconomic status (aOR: 1.86), and speaking English as a primary language in the household (aOR: 1.59) were significant predictors of knowledge of BSE. Eleven women reported ongoing breast disease symptoms resulting in an overall prevalence of 2.3 cases of breast disease symptoms per 1000 women.

**Conclusions:**

Socioeconomic disparities in access to health education may be a determinant of knowledge of BSE. Community-based strategies are needed to improve dissemination of breast cancer screening methods, particularly for women who face barriers to accessing care.

## 1. Introduction

Benign and malignant conditions of the breast are of primary concern to women's health worldwide. Breast cancer, in particular, is the most common cancer and one of the highest contributors to disability-adjusted life years (DALYs) in women. In 2013, breast cancer accounted for 464,000 deaths and 13.1 million DALYs globally, with 63% of DALYs occurring in developing countries [1]. Across Sub-Saharan Africa, the incidence and mortality of breast cancer have been continuously rising, primarily due to aging and population growth, increased urbanization, and a higher prevalence of risk factors associated with economic development (e.g., smoking, obesity, physical inactivity, and changing reproductive behaviors) [2]. This growing trend has also been observed in the central African country of Cameroon, where breast cancer is now the leading cancer among women in Yaoundé, comprising 18.5% of all cancers and 32.5% of female cancers [3]. According to the International Agency for Research on Cancer, there were 2,625 new cases of breast cancer per 100,000 women in Cameroon during 2012 [4]. This estimate, however, was based on data extrapolated from a regional hospital-based cancer registry covering part of the country [5]. Thus, currently available data may not accurately reflect the true burden and patterns of breast disease across all regions of Cameroon, especially since registries do not account for individuals who fail to present to formal care.

Despite this limitation, data from hospital-based studies indicate that a majority of breast cancers in Cameroon are diagnosed at advanced stages and result in poor outcomes [6–8]. A recent review of breast cancer data suggests that the lack of early detection programs and limited access to surgical care, the primary treatment modality for breast cancer in Sub-Saharan Africa, are likely contributors to poor overall survival among breast cancer patients in the region [9]. A retrospective cohort study conducted in Yaoundé, Cameroon, found an overall 5-year survival rate of 30% and a 10-year survival rate of 13.2% among breast cancer patients treated between 1995 and 2007 [8]. In contrast, breast cancer survival rates in high-income countries are reported to be over 80% [10–12]. This disparity highlights the critical role early detection and access to surgical treatment represent in improving breast cancer outcomes and survival, as they remain central components of breast cancer control strategies [13].

Early diagnosis of breast cancer is critical to reducing cancer-related mortality, particularly where radiation, hormonal, and chemotherapy are not widely available. Early detection relies on breast awareness and utilization of screening methods. While mammography is the only screening modality proven to reduce breast cancer mortality, mammography is neither affordable nor feasible in many low- and middle-income countries (LMICs) [14, 15]. Consequently, alternative screening methods, such as breast self-examination (BSE), have been recommended in these settings to promote breast health awareness and allow for the early detection of breast abnormalities [15]. BSE is a simple screening method that can be performed at no cost. Positive associations have been found between the practice of BSE and the detection of breast cancer, and previous studies have indicated that a majority of early-stage breast tumors are self-detected [16, 17]. Yet, the main barrier to practicing BSE has been demonstrated to be a lack of knowledge or awareness of BSE [18].

Though prior studies in the Southwest region of Cameroon have assessed knowledge and practice of BSE among small urban samples [19, 20], it is not clear whether their findings are broadly applicable to the mixed urban-rural Southwest region as a whole. Women residing in rural or difficult to access settings might be at greater risk for poor health awareness. Moreover, the burden and patterns of breast disease in Southwest Cameroon are unknown due to a lack of population level data [5]. To rectify this knowledge gap and better inform policy, a large-scale community based study is needed. The purpose of this study was to assess the knowledge and practice of BSE among women in the Southwest region of Cameroon and identify its associated factors. Additionally, this study sought to estimate the prevalence and describe patterns of breast disease symptoms among symptomatic women in the region.

## 2. Materials and Methods

### 2.1. Study Design and Setting

This cross-sectional study was conducted as a subanalysis of a larger community-based survey investigating the prevalence of injury and unmet surgical need in the Southwest region of Cameroon. This region is one of two predominantly Anglophone regions in the country, with an estimated population of 1,575,224 [21].

### 2.2. Study Population

**Figure 1:**
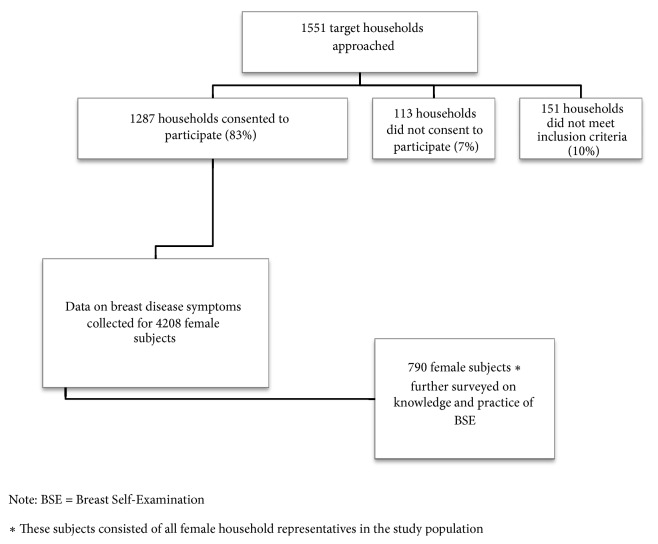
Study population selection flow chart.

The study population consisted of all female household members residing in Southwest Cameroon. Data on all female household members was collected to estimate the prevalence of breast disease symptoms in the region. Additionally, female subjects designated as household representatives were further surveyed on their knowledge and practice of BSE.  Figure 1 outlines the selection process for all study subjects included in the analysis of the prevalence of breast disease symptoms and assessment of knowledge and practice of BSE in Southwest Cameroon.

All households from whom consent was obtained were included in the study. Households without a suitable representative (a household member aged 18 years or older) present after two attempts or any individual not permanently residing in the Southwest region were excluded from the study.

### 2.3. Sampling Method and Sample Size Calculation

Enumeration areas were selected using a 3-stage clustered sampling framework. Due to safety concerns, two districts within the Southwest region (Akwaya and Bakassi) were excluded from the sampling framework. Probability proportionate to size sampling was used to select nine health districts and four health areas per district. Using geographic coordinates, a starting point was randomly selected within each health area, and contiguous households were approached from that point on until the target household number (n= 200) at each site was reached.

This study was nested in a larger community based survey whose sample size was calculated to provide 78% power to detect a 6% prevalence of injury. This prevalence estimate is based on findings from prior population-based surveys carried out in Sub-Saharan Africa [22–24]. The target sample size calculation was adjusted by a design effect of 2 to account for a loss in effectiveness and a larger variance when using a multi-clustering sampling framework, as well as a predicted 20% nonresponse rate. The minimum calculated target sample size was then deliberately exceeded by 50% to allow for multiple subanalyses of rare events. Finally, to verify that this final sample was large enough to conduct a subanalysis on breast disease, a separate sample size calculation was conducted to provide 78% power to detect a 2.9% prevalence estimate of breast disease, which was derived from a prior population-based study conducted in Sierra Leone [25].

### 2.4. Data Collection

Data collection was carried out between January 3 and March 3, 2017. Each household was asked to identify a household representative over the age of 18 years, who was then approached using a standard oral consent script. If consent was obtained, the household representative was given a pretested, context-adapted instrument based on the Surgeons OverSeas Assessment of Surgical Need (SOSAS) survey. The SOSAS survey is a tool designed to measure the prevalence of surgically treatable conditions. The validation process of the SOSAS tool, which was carried out in several developing countries, has been previously described [26, 27]. Household representatives were asked to provide information on sociodemographics, breast disease symptoms, defined as any alterations to the breast such as lumps, cancer, or skin or nipple changes or discharge, and care-seeking behaviors for female household members. Female household representatives were further asked about their own knowledge and practice of breast self-examination (BSE).

Socioeconomic status (SES) variables assessed in this study were selected based on an economic clusters model developed using nationally representative household assets data from Cameroon's Demographic and Health Survey [28]. Cooking fuel usage, more specifically the use of Liquid Petroleum Gas (LPG), has been demonstrated to correlate with higher SES in Sub-Saharan Africa, as it is a clean cooking fuel option that is prohibitively expensive for many households in the region [29, 30].

### 2.5. Statistical Analyses

All population estimates were adjusted for clustering methodology using a methodology derived from the World Health Organization's guidelines for conducting community based surveys on injuries and violence [29]. Descriptive statistics were carried out using frequencies, proportions, medians, and interquartile ranges. Comparisons between population estimates were conducted using the Adjusted Wald test and Pearson Chi Square test as appropriate. Univariate and multivariate logistic regression analyses were used to identify significant factors associated with the knowledge of BSE. A multivariate logistic regression model was built using sociodemographic and socioeconomic indicators, as well as variables relating to spoken language in the household and at health facilities. These variables were selected based on a review of prior studies investigating knowledge of BSE in LMICs [31, 32]. Covariates included in the final logistic regression model were selected using a backward stepwise regression procedure. All data were stored in REDcap, a secure online database, and were analyzed using STATA version14 [33, 34].

### 2.6. Ethical Considerations

Ethical approval for this study was obtained from the Committee for Human Research at the University of California, San Francisco, as well as the Institutional Review Board of the University of Douala.

## 3. Results

Household representatives from 1287 surveyed households provided information on 4208 female subjects. The median age of female subjects was 21 years, with a range of 0 to 115 years. Most subjects belonged to households where at least one family member had completed a secondary (36.9%) or tertiary level of education (40.6%). Over two-thirds of subjects reported residing in a rural setting (69.9%). A majority of subjects belonged to households that owned a cellular telephone (95.4%), owned agricultural land (64.2%), and used wood as a source of cooking fuel (92.4%). As compared to the overall female population of Southwest Cameroon (based on data from the 2011 Cameroon Demographic Health Survey [21]), subjects in this study belonged to households reporting a higher ownership of cellphones (95.4% vs. 70.2%) and more members with a secondary level of education or higher (77.5% vs. 38%) (Table 1).

### 3.1. Patterns of Breast Disease Symptoms and Care Seeking Behaviors

Among the 4208 study subjects, household representatives identified 11 subjects with ongoing symptoms of breast disease at the time of the survey, resulting in an overall prevalence of 2.3 cases of self-reported breast disease symptoms per 1000 women (95% CI 1.13-4.84) in the Southwest region of Cameroon. The estimated prevalence of breast disease symptoms increased to 4.02 cases per 1000 women (95% CI 0.93-17.10) when the sample population was restricted to female household representatives (N=790).

Study subjects reporting breast disease symptoms had a median age of 27 years (IQR 21, 44). Most (72.7%, n=8) reported that their symptoms had developed slowly over time for a mean duration of 7.8 years (SD±10.1). Over half of subjects with ongoing breast disease symptoms described the problem as a breast mass and over a quarter reported having non-lactation-related nipple discharge. Breast disease symptoms were predominantly unilateral and developed outside of pregnancy or lactation (Table 2).

Although 63.6% (n=7) of subjects with breast disease symptoms had sought formal care for their problem, four participants with ongoing breast symptoms had not yet presented to formal care at the time of the survey (36.4%). The reasons provided for not seeking formal care were the perception that the symptom was not serious (n=2), a lack of awareness that the symptom could be treated (n=1), and the belief that formal care was too expensive (n=1). Among the seven subjects with breast symptoms who had previously presented to formal care, four had not yet received any surgical intervention and three reported recurrent disease following an operative intervention. The most common reason provided for not obtaining surgery as a treatment option was the patient's perception that surgery was not needed (75%, n=3).

### 3.2. Knowledge and Practice of Breast Self-Examination

Information regarding the knowledge and practice of BSE was collected from all female household representatives (n=790) in the study population. A majority of these household representatives (74.6%) indicated having no knowledge of BSE. Of these, 46.3% (n=357) had never heard of BSE, while 26.7% (n=211) had previously heard of BSE but lacked the knowledge to perform it. Among women reporting knowledge of BSE (n=201, 25.4%), 16% practiced it once per year (n= 32), 56% practiced it several times per year (n=113), and 15% practiced BSE on a monthly basis (n= 30). The majority of women who practiced BSE (93.8%) did not wait to perform it at a specific time of the month, whereas a small minority performed BSE the week (3.1%) or the second week (0.3%) following their menses.

Women with knowledge of BSE were significantly older (median: 34 years vs. 31 years) and reported a higher rate of LPG usage as a source of cooking fuel as compared to women without knowledge of BSE. LPG is an indicator of higher SES. Significant differences in household education patterns were also observed between women with and without knowledge of BSE (p=0.03). A higher proportion of women with knowledge of BSE reported having a family member who achieved a tertiary level of education as compared to women without knowledge of BSE (44.4% vs. 33.1%) (Table 3).

Women without knowledge of BSE were significantly more likely to report a lack of proximity to formal medical care as a primary barrier to care (p=0.05) and these participants were also more likely to cite walking as their means of transport to formal care (50.7% vs. 42.5%). In contrast, women with knowledge of BSE were more likely to report the use of a motorized vehicle as a means of transport to access formal care (p=0.02) (Table 3).

Women with knowledge of BSE were more likely to report English as the primary spoken language in their households in addition to the language used to communicate with health providers. Conversely, women without knowledge of BSE were more likely to report the use of Pidgin English to communicate with health providers (Table 3). These participants were also less likely to believe that injuries (78.6% vs. 89.1%, p=0.03) and deformities (64.7% vs. 79.6%, p<0.01) could be treated with surgery. No significant differences were found between the two groups regarding the treatability of some cancers by surgery (63.1% vs. 63.0%, p=0.20).

A multivariate logistic regression analysis indicated that age (aOR=1.04, p<0.01), English as the primary household language (aOR=1.59, p=0.045), and LPG use (aOR=1.86, p= 0.034) were all significant independent predictors of knowledge of BSE (Table 4).

## 4. Discussion

In this study, we demonstrate that knowledge and practice of breast self-examination (BSE) is extremely poor among women in the Southwest region of Cameroon. Our findings indicate that women in this region are significantly more likely to know how to perform BSE if they use LPG as a source of cooking fuel, belong to a household where English is the primary spoken language, or are of an older age. These results are consistent with an array of previously published literature from developed countries demonstrating that higher SES, education, and age are all predictive factors of BSE knowledge and compliance [31, 32]. Although it is not possible to definitively establish why these factors associate with improved BSE knowledge in a cross-sectional survey, we can speculate that women who utilize LPG and are of a higher SES have better access to health services and more opportunities to engage with health professionals, resulting in a greater health literacy about breast screening methods. Indeed, it is common for women to receive health education lectures from health providers, particularly during antenatal care visits [35]. Conversely, participants who lacked knowledge about how to perform BSE reported increased barriers to accessing healthcare, including greater distance to healthcare facilities and use of walking as the most likely transport modality to seek care following serious injury.

Although this study did not specifically investigate sources of BSE knowledge, our conclusion that knowledge of BSE may be associated with access to healthcare may suggest that health professionals perform an important role in teaching BSE. If this is indeed the case, emphasizing provider training in clinical encounters could be exploited as a tool to increase breast-screening rates and quality in the Southwest region of Cameroon. Furthermore, the association of household English utilization with increased awareness of BSE may indicate a disproportionate dissemination of health information in English as opposed to Pidgin or other local dialects. Expanding language competency of healthcare providers in Pidgin also may hold promise as a means of increasing dissemination of BSE knowledge. Future population-based studies should investigate sources of health care knowledge regarding breast screening.

The prevalence of breast disease symptoms in this study population was lower than anticipated based on results from numerous studies suggesting women in Sub-Saharan Africa present at late stages [9]. Currently available data, though restricted in their generalizability, also point to a rising trend in breast cancer incidence in Cameroon [5]. Consequently, a community-based study was expected to identify a significant amount of undiagnosed breast disease cases across the Southwest region, given the absence of a population-based mammography-screening program and evidence demonstrating poor practice of alternative early detection methods [19, 20]. Yet, as compared to recent population-based surveys in Rwanda and Sierra Leone which detected a 4.4% and 2.9% prevalence of self-reported breast masses in women, respectively, the prevalence of self-reported breast disease symptoms found in this study was relatively low [25]. Our findings therefore call into question whether sociocultural factors, such as stigma, may have contributed to reduced reporting of breast disease symptoms among study subjects. Internalized stigma associated with breast cancer has been identified in parts of Sub-Saharan Africa [36]; however more rigorous studies are needed to better ascertain how stigma contributes to delaying cancer care engagement [37].

Surgical intervention is currently the primary modality of breast cancer treatment in Africa; and in most cases, surgical biopsy is also the means of establishing diagnosis for breast disease symptoms [9]. Yet, over a third of study participants reported that they did not believe that cancers could be surgically treated. Poor understanding of the therapeutic role of surgery in cancer care may contribute to delayed presentation or treatment among persons with breast symptoms. Although only a small number of subjects in this study were identified as having ongoing breast symptoms, those who failed to seek care or obtain surgery primarily cited a belief that their symptoms were not a serious health risk or that surgery was unnecessary. Given the high documented prevalence of late-stage presentation and poor outcomes of breast cancer in Sub-Saharan Africa [9], these findings warrant further investigation into the role of treatment perceptions in determining presentation and timing of formal-care seeking.

### 4.1. Limitations

Reliance on self-reported data, particularly from a single member of the household on behalf of other family members, is a limitation of our study. Designated household representatives may not have been aware of some breast disease symptoms in female members of their households, and representative knowledge likely varied based on family dynamics and the relationship of the representative to female family members. This factor could have led to an underestimation of the true prevalence of breast disease in the study population. To gauge the magnitude of this effect, we compared the prevalence of breast symptoms above with the prevalence of self-reported breast disease among female household representatives. Notably, restricting the sample population to female household representatives resulted in a near doubling of the estimated prevalence of breast disease. On the basis of this discrepancy in finding, we would recommend that future studies determining the prevalence of breast disease symptoms should collect data only directly from individual subjects, given the sensitive nature of the disease topic.

Importantly, it should be noted that this limitation did not affect the primary outcome of this study, as breast self-examination was only assessed among female household representatives; however, as with all survey-based data, knowledge of BSE was self-reported. Household representatives were not objectively assessed regarding their ability to correctly perform BSE, which could have potentially resulted in overestimation of BSE knowledge.

Finally, although summary data is reported for subjects identified as having ongoing breast symptoms, the size of this cohort is too small to support in-group comparisons or for extensive generalization to the larger study population.

## 5. Conclusions

Socioeconomic factors predict knowledge of BSE, raising concerns about disparities in access to health education, particularly as it pertains to women's health issues. Findings from this study emphasize the need to develop community-based strategies for improved and equitable dissemination of health information to ensure that breast cancer prevention and screening tools reach populations living in remote areas, who face greater barriers to accessing formal care. Furthermore, it is critical that this health information be communicated in a language that a majority of people in the region can easily comprehend, such as Pidgin English or local dialects in this case. Adopting alternative models to create awareness, such as community health worker interventions, could be potential solutions to spreading health information on breast cancer prevention in underserved communities.

## Figures and Tables

**Table 1:** Sociodemographic characteristics of 4208 female subjects and their households, in Southwest Cameroon.

**Characteristics**	**Frequency**
	**(**%**) or**
	**Median [IQR]**
**Age **	21 [10, 34]

**Household members **	7 [5, 10]

**Household Setting**	
Urban	1244 (30.1%)
Rural	2886 (69.9%)

**Household possesses a cellphone**	3901 (95.4%)

**Household owns agricultural land**	2650 (64.2%)

**Use of cooking fuel in household**	
Wood	3849 (92.4%)
Liquid Petroleum Gas (LPG) fuel	1823 (43.7%)
Charcoal	701 (16.8%)
Kerosene	697 (16.7%)
Other	9 (0.2%)

**Highest educational level achieved by a household member**	
No formal education	84 (2.0%)
Primary	834 (20.3%)
Secondary	1517 (36.9%)
Tertiary	1668 (40.6%)

**Household members believe some cancers can be surgically treated**	2624 (63.0%)

**Household members believe deformities can be surgically treated**	2968 (71.2%)

Note: IQR = interquartile range.

**Table 2:** Patterns of breast disease symptoms among symptomatic study subjects in Southwest Cameroon (n=11).

**Patterns **	**Frequency (**%**)**
**Symptom **	
Lump	6 (52.6%)
Abnormal breast discharge	3 (27.3%)
New asymmetry	1 (9.1%)
Other	1 (9.1%)

**Location of Symptom **	
Unilateral	6 (54.6%)
Bilateral	5 (45.5%)

**Symptom started while pregnant or breast feeding**	
Yes, while pregnant	2 (18.2%)
Yes, while breast feeding	3 (27.3%)
No	6 (54.6%)

**Progression of Symptom**	
Slowly	8 (72.7%)
Suddenly	2 (18.2%)
Unknown	1 (9.1%)

**Symptom disabling**	
Yes	2 (18.2%)
No	9 (81.8%)

**Feelings of depression or shame**	
Yes	1 (9.1%)
No	10 (90.9%)

**Breast symptom having household impact** **∗**	
Yes	8 (72.7%)
No	3 (27.7%)

**Family has spent assets, savings, or borrowed money to treat current symptom**	
Yes	8 (72.7%)
No	3 (27.7%)

**Table 3:** Comparison of female household representatives by knowledge of breast self-examination (n= 790).

	**Knowledge of BSE** ^**a**^ ** ** **(n=201)**	**No Knowledge of BSE ** **(n= 589)**	**P value** ^**b**^
**Age (median [IQR])**	34 [27, 45]	31 [24, 40]	p=0.01*∗*
**Use of Cooking Fuel in Household**			
Wood	181 (90.1%)	529 (90.7%)	p=0.99
Charcoal	45 (22.3%)	92 (15.8%)	p=0.22
Liquid Petroleum Gas (LPG) fuel	113 (56.2%)	247 (42.4%)	p=0.02*∗*
Kerosene	36 (17.9%)	102 (17.5%)	p=0.89
Other	2 (1.0%)	2 (0.3%)	p=0.38
**Highest Education level achieved by any household member**			p=0.03*∗*
No formal education	3 (1.5%)	22 (3.8%)	
Primary	31 (15.7%)	134 (23.2%)	
Secondary	76 (38.4%)	227 (39.3%)	
Tertiary	88 (44.4%)	191 (33.1%)	
**Primary barrier to seeking Formal care**			
None	69 (34.3%)	220 (37.7%)	p=0.25
Too Expensive	42 (20.9%)	104 (17.8%)	p=0.15
Inaccessible (Too Far)	5 (2.5%)	38 (6.5%)	p=0.05*∗*
Belief that treatment is ineffective	1 (0.5%)	13 (2.2%)	p=0.11
Rude or Inattentive staff	56 (27.9%)	126 (21.6%)	p=0.24
Preference for traditional medicine	1 (0.5%)	5 (0.9%)	p=0.14
Preference for faith healing	0 (0%)	4 (0.7%)	p=0.12
Other	45 (22.4%)	107 (18.4%)	p=0.33
**Transport used by household to seek formal care after Injury**			p=0.02*∗*
Walking	85 (42.5%)	288 (50.7%)	
Private car	1 (0.5%)	3 (0.5%)	
Private Motorcycle	0 (0%)	4 (0.7%)	
Taxi	27 (13.5%)	64 (11.3%)	
Mototaxi	84 (42.0%)	209 (36.8%)	
Bus	3 (1.5%)	0 (0%)	
**Primary spoken language in Household **			p=0.05*∗*
Pidgin English	57 (28.5%)	200 (34.7%)	
English	76 (38.0%)	153 (26.5%)	
French	6 (3.0%)	17 (3.0%)	
Local dialect	54 (27%)	180 (31.2%)	
Other	7 (3.5%)	27 (4.7%)	
**Primary spoken language used to communicate with health providers: **			
Pidgin English	80 (39.8%)	313 (53.7%)	p=0.03*∗*
English	143 (71.1%)	363 (62.3%)	p<0.01*∗*
French	10 (5.0%)	38 (6.5%)	p=0.37
Local dialect	4 (2.0%)	20 (3.4%)	p=0.70
Other	2 (0.3%)	1(0.5%)	p=0.55

Note: BSE = breast self-examination. An asterisk (*∗*) represents a p value of ≤ 0.05. Frequencies and percentages may not add up as a result of missing data.

^a^Knowledge of BSE indicates that the study subject reported knowing how to perform BSE on herself.

^b^All p value estimates were calculated using the adjusted Wald test and Pearson Chi Square test. The Wald test was adjusted for all variables in the data.

**Table 4:** Predictors of knowledge of breast self-examination among female household representatives in Southwest Cameroon.

**Predictor** ^**a**^	**Adjusted OR**	**95**%** CI**	**P value**
**Age** ^b^	1.04	(1.01-1.06)	p <0.01*∗*

Use of Liquid Petroleum Gas (LPG)^c^	1.86	(1.06 -3.25)	p=0.03*∗*
as cooking fuel in the household			

**Primary barrier to seeking Formal Care: Inaccessibility (Too far)** ^d^	0.33	(0.10 -1.04)	p=0.06

**English is primary spoken language in household** ^**e**^	1.59	(1.01 -2.48)	p=0.05*∗*

Note: OR = odds ratio; CI = confidence interval. An asterisk represents a p value of ≤ 0.05

^a^The final multivariable logistic regression model was built using a backward stepwise regression procedure.

^b^The reference group consisted of subjects of a younger age.

^c^The reference group consisted of subjects belonging to households where LPG was not used as a source of cooking fuel.

^d^The reference group consisted of subjects who did not report inaccessibility as a primary barrier to seeking formal care.

^e^The reference group consisted of subjects who did not primarily speak English in the household.

## Data Availability

The data used to support the findings of this study are available from the corresponding author upon request.
